# P02.197. Self help intervention to decrease stress and increase mindfulness: a pilot trial

**DOI:** 10.1186/1472-6882-12-S1-P253

**Published:** 2012-06-12

**Authors:** V Sharma, B Bauer, K Prasad, A Sood, D Schroeder

**Affiliations:** 1 Mayo Clinic, Rochester, USA

## Purpose

The Stress Management and Resiliency Training (SMART) program has shown efficacy for reducing anxiety and perceived stress and increasing resilience and quality of life. SMART has traditionally consisted of an in-person training session with an instructor and follow up teleconferences over 12-24 weeks. The purpose of this study was to assess whether self-directed SMART training using only written material would have efficacy.

## Methods

Thirty-eight subjects working at a large midwestern medical center were recruited and given a book and workbook that provided them with the background and training in the SMART program. Subjects were instructed to read through and practice the various SMART principles and techniques. These techniques included education about the neurophysiology of stress and resilience, training attention to focus on present moment awareness, refining interpretations, and cultivating higher values such as gratitude, forgiveness and higher meaning. Primary outcome measures assessed at baseline and week 12 included the Connor Davidson Resilience Scale (CDRS), Mindfulness Attention Scale (MAAS), Perceived Stress Scale (PSS), Smith Anxiety Scale (SAS), and Overall Quality of Life (QoL).

## Results

Thirty-three subjects completed the study and provided baseline and follow up data. Mean age was 48.1 years (range 27 to 66 years) and 85% of participants were female. Statistically significant improvements in resilience, mindfulness, perceived stress, anxiety, and overall QoL wereas observed at 12-weeks compared to baseline: (CDRS: 73.4±10.8 vs. 81.8±13.8, p<0.001), (MAAS: 3.7±0.7 vs. 4.3±0.9, p<0.001), (PSS: 25.7±5.6 vs. 19.5±7.3, p<0.001), (SAS: 55.5±15.4 vs. 41.7±14.9, p<0.001), and (QoL: 7.0±1.7 vs. 8.0±1.5, p=0.001).

## Conclusion

This study demonstrates that a brief, self-directed program to decrease stress and enhance resilience and mindfulness is feasible. Further, the program provides excellent short-term efficacy for enhancing resilience, mindfulness and quality of life, and decreasing stress and anxiety. Future randomized trials with larger sample sizes are warranted to study the effectiveness of the intervention.

